# A Visualization Analysis of Machine Learning Applications in Gas Adsorption Using Nanoporous Materials

**DOI:** 10.3390/nano16140883

**Published:** 2026-07-17

**Authors:** Xin Zhong, Xiong Liang, Huixia Zhang

**Affiliations:** 1 School of Physics and Mechanical and Electronical Engineering, Longyan University, Longyan 364012, China; 2 College of Life Sciences, Longyan University, Longyan 364000, China

**Keywords:** machine learning, gas adsorption, nanoporous materials, metal–organic frameworks, carbon dioxide capture

## Abstract

Machine learning has created new opportunities for gas adsorption research using nanoporous materials, but the field’s evolution remains insufficiently quantified. This study retrieved literature from the Web of Science Core Collection for 2010–2026 and retained 730 valid records from 1581 initial publications after screening. VOSviewer, CiteSpace, and R were used to analyze publication growth, collaboration networks, journal sources, and thematic evolution. Results show that annual output remained generally below 20 before 2019, then increased rapidly and reached approximately 280 publications in 2025, indicating accelerated integration of machine learning with adsorption simulation, material screening, and performance evaluation. The source distribution broadened from a limited set of chemistry and engineering journals to diverse venues, with recent high publication weights in *Chemical Engineering Journal*, *Separation and Purification Technology*, *ACS Applied Materials & Interfaces*, *Microporous and Mesoporous Materials*, and *Journal of Materials Chemistry A*. Collaboration analysis identified 10 compact author clusters, including groups associated with Randall Q. Snurr, Seda Keskin, Zhiwei Qiao, Qingyuan Yang, and Chongli Zhong, whereas the weak bridging links among clusters indicate that cross-community collaboration remains limited. Country and institutional analyses show that China, the United States, Canada, Iran, India, South Korea, and the United Kingdom are leading contributors, with Guangzhou University, Koç University, Northwestern University, the Chinese Academy of Sciences, Beijing University of Chemical Technology, and the United States Department of Energy occupying prominent positions. Keyword evolution reveals a shift from adsorption behavior and porous adsorbents toward data-guided material selection, high-throughput screening, deep learning, Bayesian optimization, and performance optimization, offering guidance for data-driven adsorbent discovery.

## 1. Introduction

Gas adsorption using nanoporous materials has become an important route for addressing problems related to carbon capture and the separation or storage of energy-relevant gases. These materials are attractive because gas molecules can enter confined pore spaces, where adsorption strength and selectivity are regulated by pore geometry and local chemical environments [1,2,3]. Metal–organic frameworks have received particular attention because their structures can be rationally adjusted through the choice of metal nodes and organic linkers [4,5,6,7,8,9]. Covalent organic frameworks, zeolites, porous aromatic frameworks, and porous carbons have also been explored as important adsorbents because they provide additional structural diversity and stability for gas adsorption applications [10,11,12]. However, the same structural diversity that makes nanoporous materials attractive also creates a difficult screening problem, since the number of possible candidates is far beyond what can be evaluated efficiently through conventional experiments.

Computational screening has helped reduce this burden by enabling large numbers of nanoporous structures to be evaluated before synthesis. Hypothetical MOF libraries and computation ready experimental databases have provided a foundation for identifying promising adsorbents under defined adsorption conditions [13,14,15]. Data-driven material design has further shown that simulation-derived information can guide the search for MOFs suitable for practical CO_2_ capture scenarios [16]. More recently, adsorption databases containing large numbers of simulated data points have made it possible to reuse computational results for broader model development [17]. Even with these advances, simulation-based screening remains sensitive to modelling choices, including how framework structures are treated and how gas framework interactions are described. As the number of materials, gases, and operating conditions increases, repeated simulation of every candidate becomes increasingly inefficient, creating a clear need for faster predictive approaches.

Machine learning has emerged as a practical strategy for improving the efficiency of nanoporous adsorbent discovery. Early studies showed that learning models could rapidly identify promising MOFs for CO_2_ capture and predict adsorption performance with much lower cost than exhaustive simulation [18,19]. Descriptor-based studies then demonstrated that model accuracy can be improved when material features are linked to physically meaningful adsorption behavior [20,21]. Subsequent work extended machine learning from individual prediction tasks to large-scale screening and gas separation analysis [22,23,24,25]. Other studies introduced pore geometry descriptors, topological representations, and graph-based models to better encode the structural information of nanoporous materials [26,27,28,29,30,31]. More recent approaches, including transformer architectures and machine learning potentials, further indicate that the field is moving toward integrated workflows in which structural representation, adsorption prediction, and molecular-level modelling are more closely connected [32,33,34,35,36,37].

Despite these advances, the use of machine learning in studies of gas adsorption by nanoporous materials remains uneven. Many studies use different data sources, descriptors, simulation conditions, and validation procedures, which makes it difficult to compare models across materials, gases, and adsorption scenarios. In addition, complex models may achieve high accuracy but provide limited physical explanation. As a result, it is often unclear whether the relationships learned by these models are consistent with adsorption theory. Recent studies have therefore emphasized that further progress depends on reliable adsorption data, models that can be interpreted, and closer links between data-driven methods and physical understanding [38,39,40,41,42]. To examine this developing field from a broader view of the literature, researchers have increasingly used quantitative review methods together with visual tools. Such approaches can show how publications increase over time, how researchers and institutions cooperate, and how research topics appear, persist, or decline. Previous studies have used these approaches to examine metal–organic frameworks in biomedical applications, carbon capture and utilization, and the use of artificial intelligence in MOF research [43,44,45]. These studies have shown that visual analysis is useful for identifying active contributors, important publication venues, major research topics, and emerging directions. However, most of them have addressed broad MOF research, specific applications, or general carbon capture technologies. A systematic visual analysis that focuses on how machine learning is used to study gas adsorption by different nanoporous materials is still lacking.

Accordingly, the study employs VOSviewer 1.6.18, CiteSpace 6.4.R1 Advanced, and R software version 4.2.3 to examine the literature and interpret the visual patterns generated from the analysis. VOSviewer was chosen because it can build and display networks that show how authors cooperate, how publications are connected through citations, and how keywords appear together [46]. CiteSpace was used to complement VOSviewer because it places greater emphasis on time. It can help identify when new topics emerge, how research fronts change, and how the knowledge base of a field develops [47]. R software was further used to calculate descriptive statistics and to generate visual outputs that could be adjusted according to the needs of this study. By combining these tools, this work examines how publication output has changed, how cooperation is organized, where related studies have been published, how keywords are connected, and how the main topics have evolved. Through this analysis, the study aims to clarify the development of research on machine learning in gas adsorption by nanoporous materials and to provide guidance for future data-driven adsorbent discovery.

## 2. Methods

This study systematically investigates the application of machine learning in gas adsorption using nanoporous materials, with the aim of clarifying research progress, collaboration patterns, topic distribution, and thematic evolution in this interdisciplinary field. Literature records were retrieved from the Web of Science Core Collection, which provides standardized records and citation information for multidisciplinary scientific research. The search was conducted on 7 May 2026, and the time span was limited to 2010 to 2026. Only publications written in English were included. To ensure consistency with the research scope, the retrieval strategy was designed to simultaneously cover machine learning methods, gas adsorption-related processes, and nanoporous material systems. The specific Topic search formula was as follows: TS = ((“machine learning” OR “deep learning” OR “artificial intelligence” OR “neural network” OR “random forest” OR “support vector machine” OR “Gaussian process” OR “graph neural network” OR “active learning” OR “materials informatics” OR “data-driven” OR “data driven” OR “Bayesian optimization” OR “high-throughput screening” OR “surrogate model” OR “inverse design” OR “explainable artificial intelligence” OR “interpretable machine learning”) AND (“gas adsorption” OR “adsorption isotherm” OR “adsorption capacity” OR “adsorption selectivity” OR “gas separation” OR “separation selectivity” OR “gas storage” OR “carbon capture” OR “CO_2_ capture” OR “carbon dioxide capture” OR “CO_2_ adsorption” OR “carbon dioxide adsorption” OR “CH_4_ adsorption” OR “methane adsorption” OR “H_2_ storage” OR “hydrogen storage” OR “Henry constant” OR “gas diffusion” OR “diffusion coefficient” OR “molecular simulation” OR “Grand Canonical Monte Carlo” OR GCMC OR “adsorption mechanism”) AND (“nanoporous material” OR “microporous material” OR “mesoporous material” OR “porous material” OR “porous framework” OR “metal-organic framework” OR “metal organic framework” OR MOF OR “zeolitic imidazolate framework” OR ZIF OR “covalent organic framework” OR COF OR zeolite* OR “porous carbon” OR “activated carbon” OR “carbon molecular sieve”)).

The initial search yielded 1581 records. To make the construction of the dataset more transparent, the retrieved records were screened in two main steps. First, the document type was restricted to Articles, Review Articles, and Proceedings Papers. Records that usually provide limited original research content, such as editorial notes, letters, corrections, meeting abstracts, book chapters, and news items, were excluded. This step helped retain publications that contained sufficient information for subsequent visual and quantitative analysis. The remaining records were then manually examined to determine whether they were consistent with the scope of this study. During this process, the title, abstract, author keywords, and Keywords Plus of each record were reviewed. When the relevance of a record was unclear, the full text was checked. A record was retained only when it connected machine learning or related data-driven methods with gas adsorption, gas separation, gas storage, or closely related adsorption processes involving nanoporous or porous adsorbents. Records were excluded when they only mentioned machine learning without applying it to adsorption analysis, when they focused on topics outside gas adsorption, or when the materials studied were not relevant to nanoporous adsorbents. After document-type restriction and manual relevance screening, 730 valid records were retained for further analysis.

**Figure 1:**
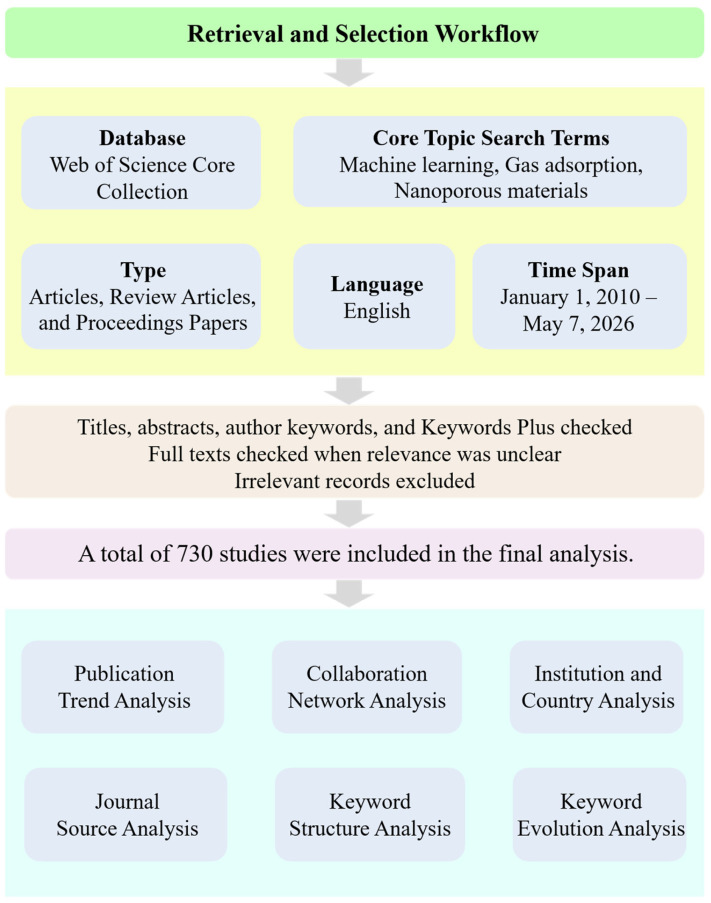
Literature retrieval, screening, and analytical workflow.

The final dataset was exported in plain text format with full records and cited references to support subsequent visualization and interpretation. On this basis, VOSviewer, CiteSpace, and R software were jointly used for data visualization and analytical interpretation. Together, these tools enabled a structured examination of research development and thematic changes in machine learning applications in gas adsorption using nanoporous materials. Figure 1 summarizes the research workflow by showing how the records were retrieved and screened before being used for visualization-based analysis. The specific parameter settings used for each visualization are reported in the corresponding analysis sections.

## 3. Results

### 3.1. Annual Trends in Publication Output

**Figure 2:**
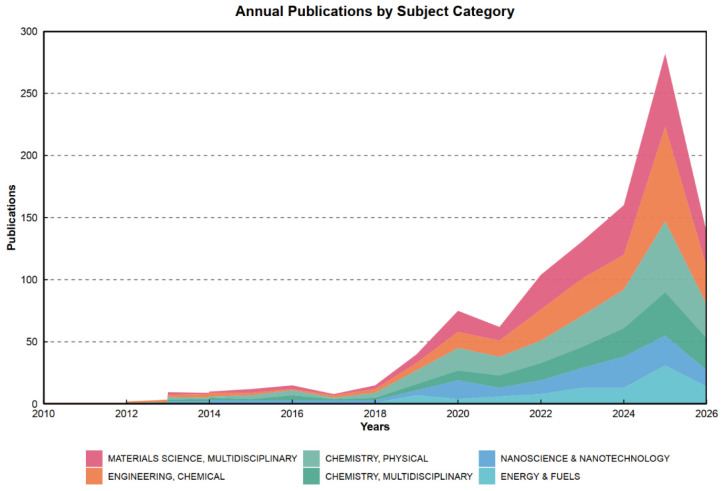
Annual trends in publications by subject category.

The annual publication trend was calculated in R using the publication year and Web of Science subject-category fields of all 730 valid records. No minimum occurrence threshold was applied to the annual output analysis. Therefore, all retained records were included. Subject categories were analyzed using the full counting method, meaning that a single publication assigned to multiple Web of Science categories could contribute to more than one category. Because the search was conducted on 7 May 2026, the publication output for 2026 should be interpreted as a partial-year result. The annual publication output on the application of machine learning to gas adsorption in nanoporous materials exhibits a clear transition from a low-activity stage to a rapid expansion stage, as shown in Figure 2. During 2010–2018, the number of publications remained limited, suggesting that machine-learning-assisted adsorption studies were still largely exploratory and had not yet become a mainstream methodological framework in nanoporous materials research. A pronounced inflection point appears after 2019, when the annual output began to increase rapidly, indicating the accelerated integration of data-driven modelling with adsorption simulation, material screening, and gas separation research.

A more detailed inspection of the subject-category distribution reveals that this growth is not driven by a single discipline but by the convergence of several research domains. Materials Science, Multidisciplinary and Engineering, Chemical constitute the major contributors, implying that the field has moved beyond purely computational prediction toward material design, process-oriented separation, and adsorbent performance optimization. The persistent contribution from Chemistry, Physical and Chemistry, Multidisciplinary suggests that molecular-level adsorption mechanisms, thermodynamic descriptors, and structure–property relationships remain central to model development. Meanwhile, the increasing presence of Energy & Fuels and Nanoscience & Nanotechnology indicates that machine learning is increasingly being applied to application-driven topics such as CO_2_ capture, hydrogen storage, methane adsorption, and energy-related gas separation using MOFs, COFs, zeolites, activated carbons, and other nanoporous adsorbents.

Notably, the sharp rise in 2025 reflects the maturation of this research direction, where machine learning is no longer used only as a statistical prediction tool but is increasingly coupled with high-throughput screening, molecular simulation, descriptor engineering, and materials informatics. This disciplinary expansion suggests a methodological shift from conventional trial-and-error adsorbent evaluation toward data-driven discovery and optimization of nanoporous materials for targeted gas adsorption applications.

### 3.2. Author Collaboration Network

To characterize author collaboration patterns, an author-level co-authorship network was constructed in VOSviewer. Co-authorship was selected as the analysis type, with authors as the unit of analysis. Authors with at least five documents were included, and no citation threshold was applied to preserve sufficient collaboration information. Full counting and association strength normalization were used. VOSviewer’s built-in clustering algorithm was then applied to identify author communities. No additional pruning was conducted, so all links among eligible authors were retained.

**Figure 3:**
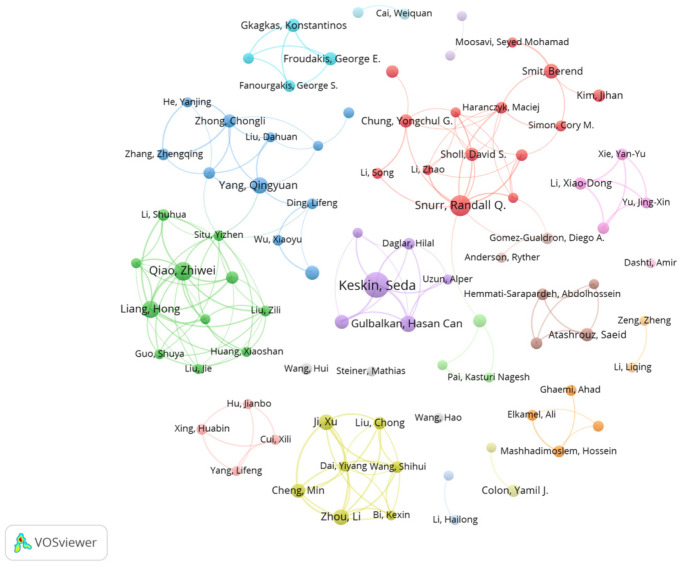
Collaboration structure among contributing authors.

The author collaboration network indicates that research on machine learning applications in gas adsorption using nanoporous materials is organized around several relatively independent scholarly communities rather than a single cohesive collaboration system (Figure 3). The presence of multiple compact clusters suggests that knowledge production in this field is largely driven by established research teams with strong internal collaboration. Groups associated with Randall Q. Snurr, Seda Keskin, Zhiwei Qiao, Qingyuan Yang, and Chongli Zhong occupy visually prominent positions in the network, reflecting their high collaboration visibility within the mapped dataset. Beyond the identification of major author communities, the network also reveals an uneven distribution of collaboration prominence. A small number of authors appear as local hubs, while many others are positioned at the periphery with fewer direct links. The absence of strong bridging connections among separated communities suggests that few authors currently play an obvious intermediary role between different collaboration groups. This topology indicates that the field has developed through parallel collaborative trajectories, and future cooperation among dispersed author groups may contribute to a more cohesive scholarly network.

### 3.3. Institutional Collaboration Network

**Figure 4:**
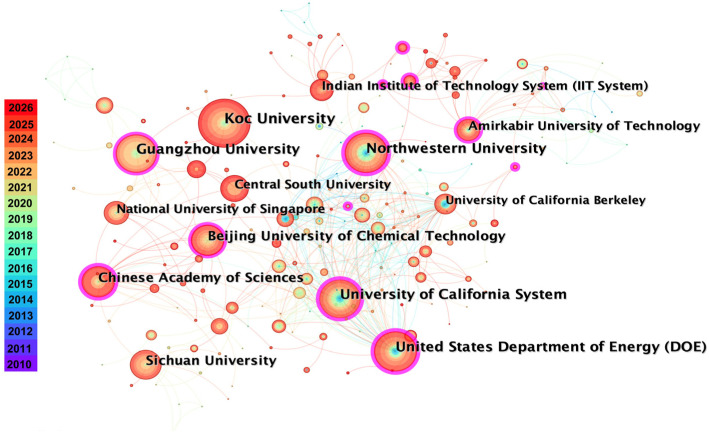
Institutional collaboration network of research organizations.

The institutional collaboration network was constructed using CiteSpace, with Institution selected as the node type. Nodes were selected using the g-index criterion (k = 25), and the network was processed using Pathfinder pruning and full counting. Institutions were connected when they appeared in the same publication. The resulting network included 261 institutions and showed low overall density but moderate clustering, indicating a broadly distributed yet only moderately integrated collaboration structure. To further quantify the institutional roles shown in Figure 4, Table 1 lists the top 15 contributing institutions ranked by frequency, together with their betweenness centrality and first appearance year.

As shown in Figure 4 and Table 1, several institutions occupy prominent positions in the network, including Guangzhou University, Koç University, Northwestern University, Chinese Academy of Sciences, Beijing University of Chemical Technology, University of California System, and United States Department of Energy. The relatively large node sizes of these institutions indicate their high visibility within the mapped collaboration network. Meanwhile, the dense links around the central region suggest that institutional cooperation in this field is not dominated by a single organization, but is distributed across multiple active institutional groups.

The overlay colors indicate the temporal distribution of institutional activity, showing that many major nodes are concentrated in the recent period. This suggests that institutional participation in machine-learning-assisted gas adsorption research has intensified in recent years. The network also shows both regional aggregation and international connection: Chinese institutions such as Guangzhou University, Chinese Academy of Sciences, and Sichuan University form visible collaborative presences, while institutions such as Koç University, Northwestern University, Amirkabir University of Technology, and Indian Institute of Technology System indicate broader international involvement. Overall, the network reflects an expanding and increasingly internationalized institutional landscape for machine learning applications in gas adsorption using nanoporous materials.

### 3.4. Country Collaboration Network

The country-level collaboration network was analyzed using R software to characterize patterns of international cooperation in this field. A country co-occurrence matrix was constructed from the 730 valid records using the full-counting method, whereby each country was counted once per publication and each country pair was counted once when both countries appeared in the same record. The chord diagram was restricted to a predefined set of 28 countries, and all country pairs with at least one co-occurrence were included. In the diagram, sector size represents the number of records associated with each country, whereas ribbon width reflects the strength of collaboration between country pairs after rescaling for visualization.

**Figure 5:**
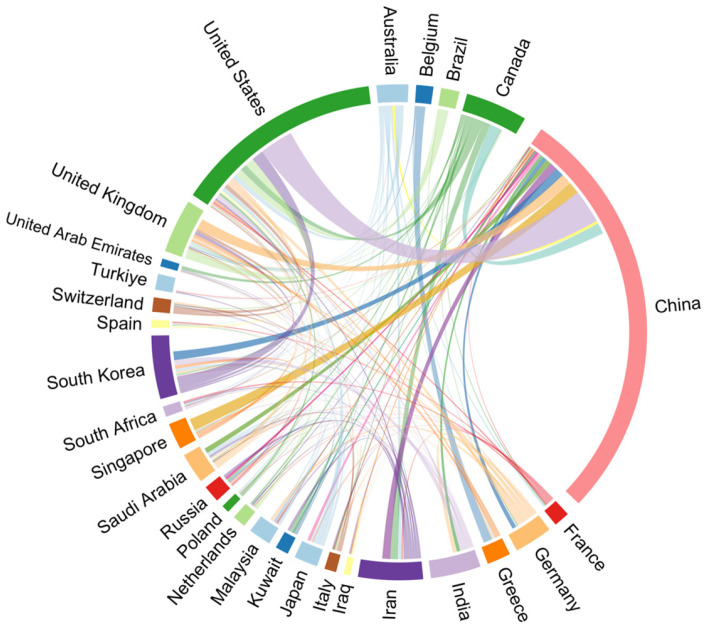
Country collaboration network for machine learning applications in gas adsorption using nanoporous materials.

The country collaboration chord diagram depicts the international cooperation landscape of research on machine learning applications in gas adsorption using nanoporous materials (Figure 5). The sector arc lengths indicate an uneven distribution of national participation, with China, the United States, Canada, Iran, India, South Korea, and the United Kingdom forming relatively visible segments. In addition, countries such as Singapore, Germany, France, Russia, the Netherlands, Malaysia, and Australia are also represented in the diagram, showing that this research topic involves contributors from Asia, North America, Europe, the Middle East, and Oceania.

The ribbon pattern is characterized by several concentrated cooperation bundles rather than a diffuse set of evenly distributed links. The most visible ribbons connect larger sectors with recurrent partner countries, particularly among China, the United States, Canada, South Korea, and the United Kingdom. Countries with intermediate sector sizes, such as Canada, South Korea, and Singapore, are linked to more than one regional group, indicating that their collaboration roles extend beyond bilateral cooperation. This configuration suggests that international collaboration in machine learning applications for gas adsorption using nanoporous materials is structured around several dense cooperation routes, with intermediate contributors connecting different parts of the country network.

### 3.5. Analysis of Journal Sources

**Figure 6:**
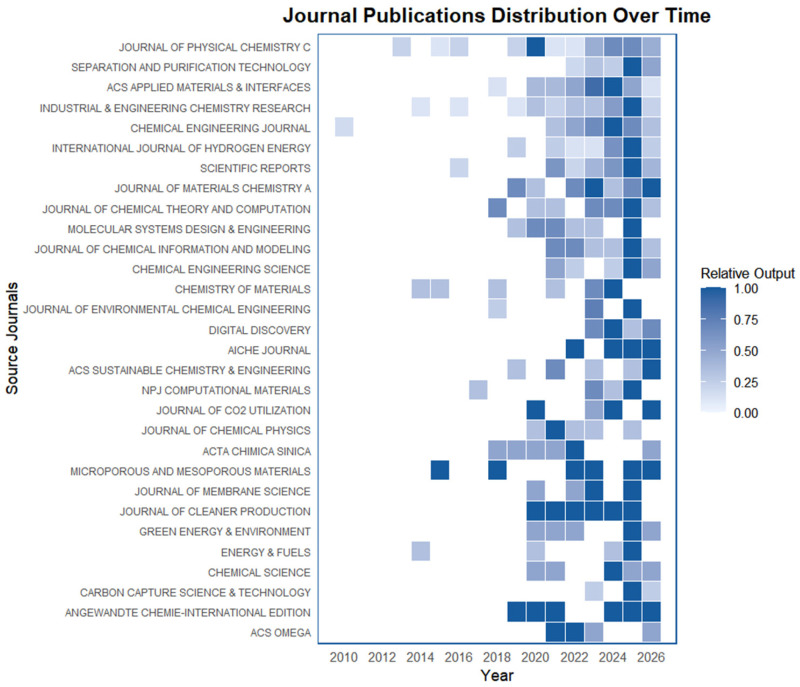
Temporal distribution of journal publication weights in ML-assisted gas adsorption.

The temporal distribution of journal sources was analyzed using R software based on the cleaned records. Source titles were first standardized to reduce inconsistencies caused by journal-name variants. Full counting was applied, and each publication was counted once according to its source journal and publication year. To improve readability, the heatmap was constructed using the 30 most productive source journals in the final dataset. In Figure 6, “relative output” was defined as the annual publication count of a given journal divided by the maximum annual publication count of the same journal during the study period. Therefore, the normalization was performed within each journal across years, rather than across journals or within a single year. A relative output value of 1 indicates the year in which that journal reached its own maximum annual output, whereas a value of 0 indicates that no relevant publication from that journal was recorded in that year. Since this journal-wise normalization emphasizes temporal changes within each journal rather than absolute productivity across journals, Table 2 provides the total publication counts of the top 20 journals for comparison.

The heatmap presents the temporal distribution of journal publications on machine learning-assisted gas adsorption in nanoporous materials. During 2010–2017, relevant publications were sparse and scattered across a limited set of journals, primarily in physical chemistry, materials chemistry, and chemical engineering. This distribution indicates that the field was still in an exploratory phase, with early studies mainly connected to conventional adsorption research, molecular simulation, and fundamental investigations of porous adsorbents.

A clear increase in publication weight is observed after 2018, with a pronounced concentration of activity from 2020 onward. High relative outputs are found in journals such as Chemical Engineering Journal, Separation and Purification Technology, ACS Applied Materials & Interfaces, Industrial & Engineering Chemistry Research, Microporous and Mesoporous Materials, and Journal of Materials Chemistry A. From 2023 to 2026, the heatmap shows a broader distribution of high-weight publication cells across energy, environmental, separation, and computational materials journals. Notably, several journals exhibit strong publication weights only in the most recent years, indicating that the recent growth of this topic is accompanied by an expansion of publication venues rather than being confined to a small group of long-standing core journals.

### 3.6. Keyword Co-Occurrence Network and Density Visualization

The keyword co-occurrence network and density map were constructed using VOSviewer to identify the conceptual structure and research hotspots of machine-learning-assisted gas adsorption research using nanoporous materials. Keywords were standardized before analysis to merge synonymous or spelling-variant terms, such as “CO_2_ capture” and “carbon dioxide capture”, and “machine learning” and “machine-learning”. The full-counting method was applied, meaning that each keyword appearing in a publication was counted once, and fractional counting was not used. A minimum occurrence threshold of 3 was set to retain frequently occurring terms for visualization. Keyword links were generated when two terms co-occurred in the same record, and the link strength represented their co-occurrence frequency. The network was normalized using the association-strength method and clustered using the VOSviewer clustering algorithm. No time slicing or pruning was applied in the VOSviewer keyword analysis. The density visualization was generated from the same keyword co-occurrence network, with color intensity reflecting the local concentration of high-occurrence and strongly connected keywords.

**Figure 7:**
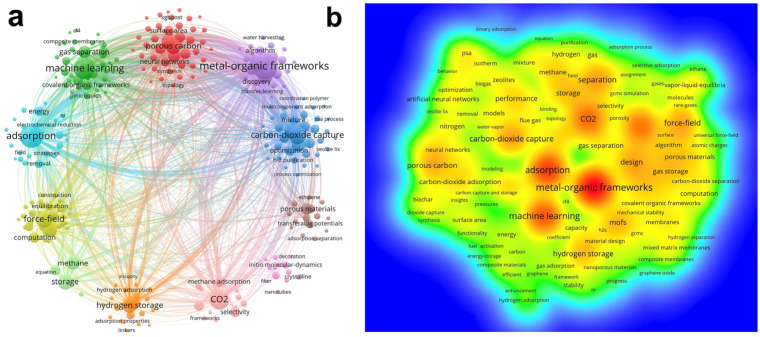
Keyword structure and hotspot distribution of machine-learning-assisted gas adsorption research. (**a**) Keyword co-occurrence network. (**b**) Keyword density visualization.

As shown in Figure 7a, the close links among machine learning, adsorption, and metal–organic frameworks indicate that data-driven methods are mainly embedded in the evaluation and optimization of porous adsorbents. The network further extends toward CO_2_, gas capture, and gas separation, suggesting that carbon capture and separation remain the most visible application contexts. Terms located near force field and computation show that computational modelling continues to provide an important basis for interpreting adsorption behavior, while terms such as discovery and transfer learning indicate the growing use of learning-based methods for material identification and performance prediction. Other branches related to hydrogen storage, porous carbon, methane adsorption, and multicomponent adsorption suggest that the application scope has expanded beyond a single gas system.

The density map (Figure 7b) supports this interpretation by showing where keyword activity is most concentrated. The highest density regions appear around metal–organic frameworks, adsorption, machine learning, CO_2_, and carbon dioxide capture, confirming that porous framework materials, adsorption performance, and carbon capture form the central focus of the field. In contrast, the lower density regions around force field, computation, hydrogen storage, methane adsorption, and multicomponent adsorption indicate supporting or developing directions rather than separate dominant centers. Taken together, Figure 7a,b show that research on machine learning applications in gas adsorption has developed around a central connection among nanoporous material design, adsorption applications, and computational prediction, while gradually extending toward broader gas systems and more diverse modelling strategies.

These keyword patterns indicate that different gas-adsorption applications occupy distinct positions within the thematic structure of the field. As shown in Figure 7, CO_2_-related terms, including CO_2_, carbon dioxide capture, and gas capture, are closely associated with the high-density core formed by machine learning, adsorption, and metal–organic frameworks. This distribution indicates that CO_2_ capture remains the most prominent application context in machine-learning-assisted gas adsorption research. Gas separation also appears as an important application-oriented topic within the keyword network. By contrast, hydrogen storage, methane adsorption, and multicomponent adsorption are located in relatively lower-density regions, suggesting that these topics currently represent supporting or developing directions rather than independent dominant research centers. Therefore, the keyword structure reveals a CO_2_-centered research core together with an outward expansion toward broader gas-storage and separation scenarios. This uneven thematic distribution highlights the need for future studies to distinguish application-specific descriptors, target properties, and model-evaluation strategies when developing machine-learning models for nanoporous adsorbent discovery.

### 3.7. Keyword Timeline Analysis

**Figure 8:**
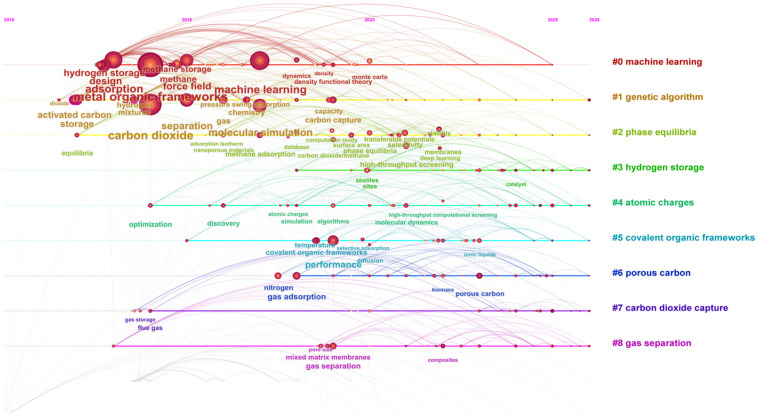
Keyword timeline of ML-assisted gas adsorption.

To capture the temporal evolution of research topics, a keyword timeline analysis was performed using CiteSpace (Figure 8). Keywords were used as the node type, with the time span set from 2010 to 2026 and a one-year time slice. Instead of manually imposing a fixed minimum occurrence threshold, representative keyword nodes in each slice were selected using the g-index criterion with k = 25. Link strengths were normalized by the cosine method, and Pathfinder pruning together with pruning of sliced networks was applied to simplify the network structure and highlight the main temporal trajectories. As a quantitative supplement to the keyword timeline map, Table 3 summarizes the main high-frequency keywords identified in the timeline analysis.

As shown in Figure 8 and Table 3, the keyword timeline map reveals a gradual shift in the thematic focus of machine-learning-assisted gas adsorption research using nanoporous materials. In the earlier stage, the map is dominated by terms linked to adsorption performance, porous adsorbents, and gas storage, indicating that the field initially relied heavily on established adsorption research. As the timeline moves forward, more recent nodes are associated with prediction, optimization, screening, and algorithm-based analysis, suggesting that the research focus has gradually shifted from describing adsorption behavior to using data-driven methods to guide material evaluation and selection.

Several thematic lines continue across different periods, showing that recent studies remain closely connected to earlier adsorption problems rather than forming an entirely separate research direction. Topics related to carbon capture, hydrogen storage, gas separation, and adsorption modelling persist throughout the timeline, while newer terms such as high-throughput screening, deep learning, transfer learning, and Bayesian optimization appear in later years. This pattern indicates a gradual methodological transition in which machine learning is increasingly used to refine existing adsorption research questions and improve the efficiency of nanoporous material discovery.

### 3.8. Keyword Thematic Evolution

**Figure 9:**
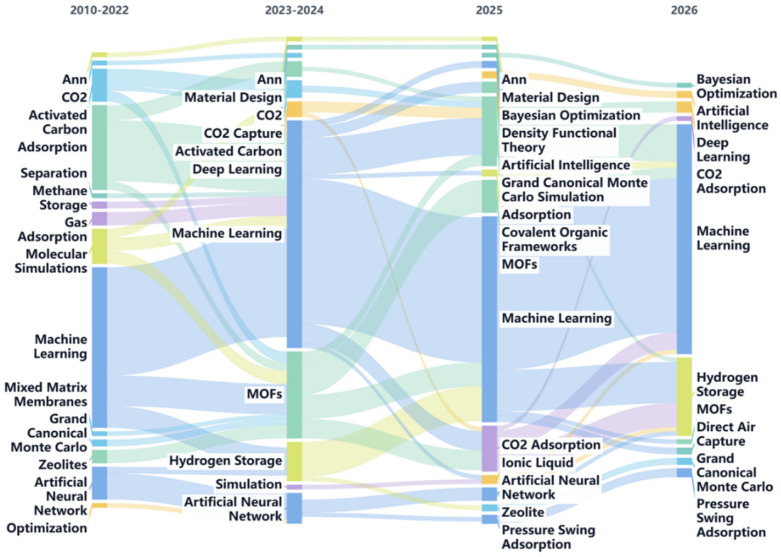
Evolutionary trends in research themes.

The R-based Sankey diagram was constructed to visualize the thematic evolution of machine-learning-assisted gas adsorption research across four periods: 2010–2022, 2023–2024, 2025, and 2026 as a partial year (Figure 9). The analysis was based on standardized combined keywords extracted from the 730 valid records. Full counting was applied, meaning that each keyword was counted once within a record, whereas fractional counting was not used. The thematic-transition table contained 70 cross-period links, including 25 links from 2010–2022 to 2023–2024, 26 links from 2023–2024 to 2025, and 19 links from 2025 to 2026. Links between adjacent periods were established according to shared keywords and were quantified using the weighted inclusion index, inclusion index, keyword occurrences, and stability index.

The thematic flows reveal a gradual shift from conventional adsorption-related topics toward data-driven material discovery and process optimization, as shown in Figure 9. In the early stage, the field was mainly focused on conventional porous adsorbents, such as activated carbon, zeolites, and mixed-matrix membranes, as well as general gas adsorption, separation, methane storage, hydrogen storage, and molecular simulation. Machine learning was already present but primarily served as a complementary tool for adsorption-property prediction and material screening.

From 2023 onward, machine learning became the central methodological framework and was increasingly coupled with metal–organic frameworks, CO_2_ adsorption, deep learning, artificial neural networks, and high-throughput simulations. The emergence of Bayesian optimization, density functional theory, grand canonical Monte Carlo simulation, pressure swing adsorption, direct air capture, and COFs indicates a shift from simple performance prediction toward integrated material discovery and process optimization. Overall, the figure highlights the convergence of machine learning, nanoporous material design, and adsorption technologies, with CO_2_ adsorption remaining the dominant application.

## 4. Conclusions

This study examined the development of machine-learning-assisted gas adsorption research involving nanoporous materials based on 730 records published between 2010 and 2026. The results show that the field moved from an early exploratory stage to rapid growth after 2019. This transition suggests that machine learning is increasingly being incorporated into workflows that combine adsorption simulation with material evaluation and adsorbent optimization. The collaboration results further show that several active research communities have formed, but links among them remain limited. Stronger cross-community cooperation would help researchers share data more effectively, compare models under clearer conditions, and support experimental validation.

Future work should move beyond the pursuit of higher prediction accuracy alone. A key priority is to build benchmark adsorption datasets in which materials, gases, operating conditions, and performance information are reported in a consistent way. Such datasets would allow different models to be evaluated under comparable conditions. Uncertainty analysis should also be incorporated so that researchers can judge when model predictions are reliable and when additional simulation or experimental verification is needed. In addition, models should be tested more carefully when they are applied to gases, material families, or adsorption conditions that differ from those used during training.

Future studies should also make model inputs more physically meaningful. Descriptors should be linked more closely to pore structure, surface chemistry, adsorption energetics, and thermodynamic behavior, so that prediction results can be interpreted in relation to adsorption mechanisms. Prediction tasks should further move from ideal single-component adsorption to more realistic conditions, including gas mixtures, humid environments, and materials that may become unstable in the presence of water. Model evaluation should also consider whether an adsorbent can deliver useful working capacity, maintain selectivity under operating conditions, be regenerated efficiently, and reduce energy demand. Experimental validation remains essential for confirming whether machine-learning-predicted adsorbents can achieve their expected performance in practical adsorption and separation processes.

The scope of this study also has limitations. The dataset was obtained only from the Web of Science Core Collection. Although this database provides standardized publication and citation information, it does not include all relevant studies. As a result, the choice of database may affect how the scale of the field, the visibility of contributors, the structure of collaboration, and the distribution of research topics are represented. The findings may also change if the search is updated, if different search terms are used, or if visualization settings are adjusted. Future visualization and scientometric studies could address these uncertainties by comparing multiple databases, updating the dataset as new publications appear, and combining analysis of publication metadata with detailed full-text examination. Such work would provide a clearer understanding of how models are developed, what data they rely on, which adsorption systems they address, and how their predictions are validated.

## Figures and Tables

**Table 1:** Top 15 contributing institutions in machine-learning-assisted gas adsorption research.

Institute	Frequency	Centrality	First Year
Koc University	33	0.04	2018
United States Department of Energy	27	0.23	2014
Northwestern University	27	0.25	2013
University of California System	26	0.30	2014
Guangzhou University	26	0.15	2018
Chinese Academy of Sciences	21	0.24	2018
Beijing University of Chemical Technology	21	0.12	2020
Sichuan University	20	0.06	2020
Central South University	18	0.03	2022
National University of Singapore	16	0.14	2016
Amirkabir University of Technology	15	0.09	2016
Indian Institute of Technology System	15	0.05	2020
University of California Berkeley	14	0.07	2014
Tianjin University	12	0.09	2022
Lawrence Berkeley National Laboratory	11	0.03	2014

**Table 2:** Publication counts of the top 20 source journals.

Journals	Total Publications
Journal of Physical Chemistry C	38
Separation and Purification Technology	36
ACS Applied Materials & Interfaces	31
Industrial & Engineering Chemistry Research	30
Chemical Engineering Journal	22
International Journal of Hydrogen Energy	21
Scientific Reports	17
Journal of Materials Chemistry A	14
Journal of Chemical Theory and Computation	12
Molecular Systems Design & Engineering	10
Journal of Chemical Information and Modeling	10
Chemical Engineering Science	10
Chemistry of Materials	9
Journal of Environmental Chemical Engineering	8
Digital Discovery	8
Aiche Journal	8
ACS Sustainable Chemistry & Engineering	8
NPJ Computational Materials	8

**Table 3:** Top 15 high-frequency keywords in machine-learning-assisted gas adsorption research.

Keyword	Frequency	Centrality	First Year
Metal–organic frameworks	372	0.02	2014
Carbon dioxide	301	0.05	2014
Machine learning	219	0	2017
Adsorption	205	0.03	2013
Molecular simulation	135	0.02	2017
Force field	124	0.04	2015
Separation	116	0.02	2015
Design	111	0.04	2013
Hydrogen storage	96	0.04	2013
Storage	89	0.03	2012
Performance	79	0.04	2019
Activated carbon	77	0.15	2012
Capture	75	0.03	2013
Prediction	71	0.03	2014
Methane	64	0.05	2015

## Data Availability

The original contributions presented in this study are included in the article. Further inquiries can be directed to the corresponding author.
